# Nursing Care of Heart Failure With Preserved Ejection Fraction: Review

**DOI:** 10.31083/RCM47695

**Published:** 2026-04-17

**Authors:** Xiaodan Feng, Haipeng Liu, Xiaomi Wang, Mengxi Xu, Linlin Ma, Lingling Zhu, Xinhong Wang

**Affiliations:** ^1^Nursing Department, The Second Affiliated Hospital, Zhejiang University School of Medicine, 310009 Hangzhou, Zhejiang, China; ^2^Centre for Intelligent Healthcare, Coventry University, CV1 5RW Coventry, UK; ^3^Department of Radiology, The Second Affiliated Hospital, Zhejiang University School of Medicine, 310009 Hangzhou, Zhejiang, China

**Keywords:** heart failure with preserved ejection fraction, nursing, pathophysiology, epidemiology, comorbidities

## Abstract

The incidence of heart failure with preserved ejection fraction (HFpEF) has been steadily increasing in recent years, which poses significant challenges to clinical management. Indeed, these rises are attributable to the incompletely understood pathophysiology of the condition, limited treatment options, a prolonged clinical course, a substantial economic burden, and frequent, complex comorbidities. Thus, to address these challenges, this review synthesizes current research on the pathophysiological mechanisms, epidemiological characteristics, and clinical management of HFpEF. Meanwhile, this review further examines the existing gaps and challenges in nursing practices, aiming to inform and optimize clinical nursing care for this patient population. A systematic literature search was performed across four databases (PubMed, Cochrane Library, Web of Science, and Embase) from inception to April 2024, using the keywords “heart failure with preserved ejection fraction”, “HFpEF”, and “nurs*”. Ultimately, after duplicates and irrelevant records were removed, 72 articles were included in this review. Nursing care plays a pivotal role in enhancing the quality of life and improving clinical outcomes for patients with heart failure with preserved ejection fraction. Future efforts should prioritize optimizing treatment plans, reinforcing patient education, developing robust risk prediction models, and exploring innovative nursing care frameworks. Additionally, attention must be paid to the rational allocation and efficient use of nursing resources to deliver more effective, individualized patient care. Current clinical practice lacks standardized nursing protocols for the diagnosis, treatment, and long-term management of HFpEF. Therefore, conducting comprehensive patient assessments, implementing evidence-based nursing interventions tailored to individual diagnoses, and integrating advanced nursing models into routine practice are essential to enhance the quality of care.

## 1. Introduction

According to the 2021 European Society of Cardiology (ESC) Guidelines for the diagnosis and management of heart failure, heart failure is not a single pathological diagnosis, but a clinical syndrome characterized by cardinal symptoms (e.g., breathlessness, ankle swelling, and fatigue) that may be accompanied by signs (e.g., elevated jugular venous pressure, pulmonary crackles, and peripheral edema). It results from structural and/or functional abnormalities of the heart that lead to elevated intracardiac pressures and/or inadequate cardiac output at rest or during exercise. Heart failure is classified into different phenotypic categories based on left ventricular ejection fraction (LVEF), namely heart failure with preserved, mildly reduced, and reduced ejection fraction. A reduced LVEF is defined as less than 40%, indicating significantly impaired left ventricular systolic function, and is classified as heart failure with reduced ejection fraction (HFrEF). Patients with an LVEF between 41% and 49% are considered to have mildly impaired systolic function and are classified as having heart failure with mildly reduced ejection fraction (HFmrEF). The diagnosis of heart failure with preserved ejection fraction (HFpEF) applies to individuals with signs and/or symptoms of heart failure, evidence of structural and/or functional cardiac abnormalities or elevated natriuretic peptides (NPs), and an LVEF of 50% or greater [1, 2]. HFpEF represents the most common subtype of heart failure, accounting for over 50% of all cases, and its prevalence has been rising in recent years. This trend is closely associated with extended life expectancy and the growing burden of related comorbidities [1, 3, 4].

Despite its increasing prevalence, the understanding of HFpEF remains incomplete. Patients are often asymptomatic in the early stages, leading to late diagnosis [5]. The pathophysiology of the condition is not fully elucidated, and specific, effective treatments are still lacking [6]. These limitations contribute to several unmet challenges in the clinical nursing of HFpEF, including uncertainty in management and potential inequities in healthcare [7]. The development of risk prediction models and optimal nursing management frameworks for HFpEF is still in the exploratory phase.

**Fig. 1.**
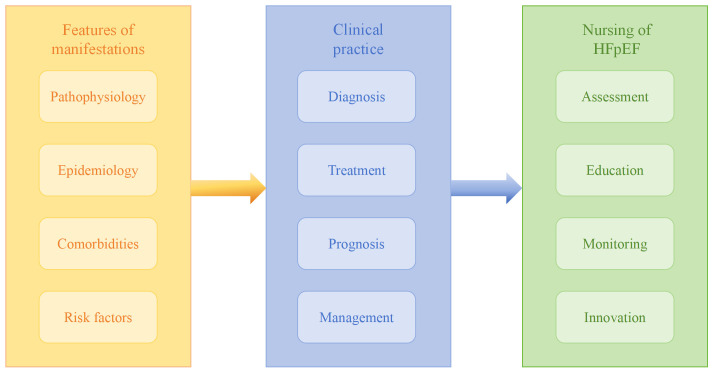
**Content summary of this article**. HFpEF, heart failure with preserved ejection fraction.

To better understand and address the unmet clinical needs in HFpEF nursing, this review synthesizes recent literature and summarizes research progress in two key aspects: clinical manifestations and nursing practice, with the aim of providing guidance for clinical nursing care. An overview of the article’s structure is provided in Fig. 1.

## 2. Literature Screening Process

In April 2024, a systematic literature search was performed across four databases: PubMed, Cochrane Library, Web of Science, and Embase. The search utilized the keywords “heart failure with preserved ejection fraction”, “HFpEF” and “nurs*” without date restrictions, and was conducted across all fields with English as the default language. The search logic applied was: ((“heart failure with preserved ejection fraction”) OR (HFpEF)) AND (nurs*).

**Fig. 2.**
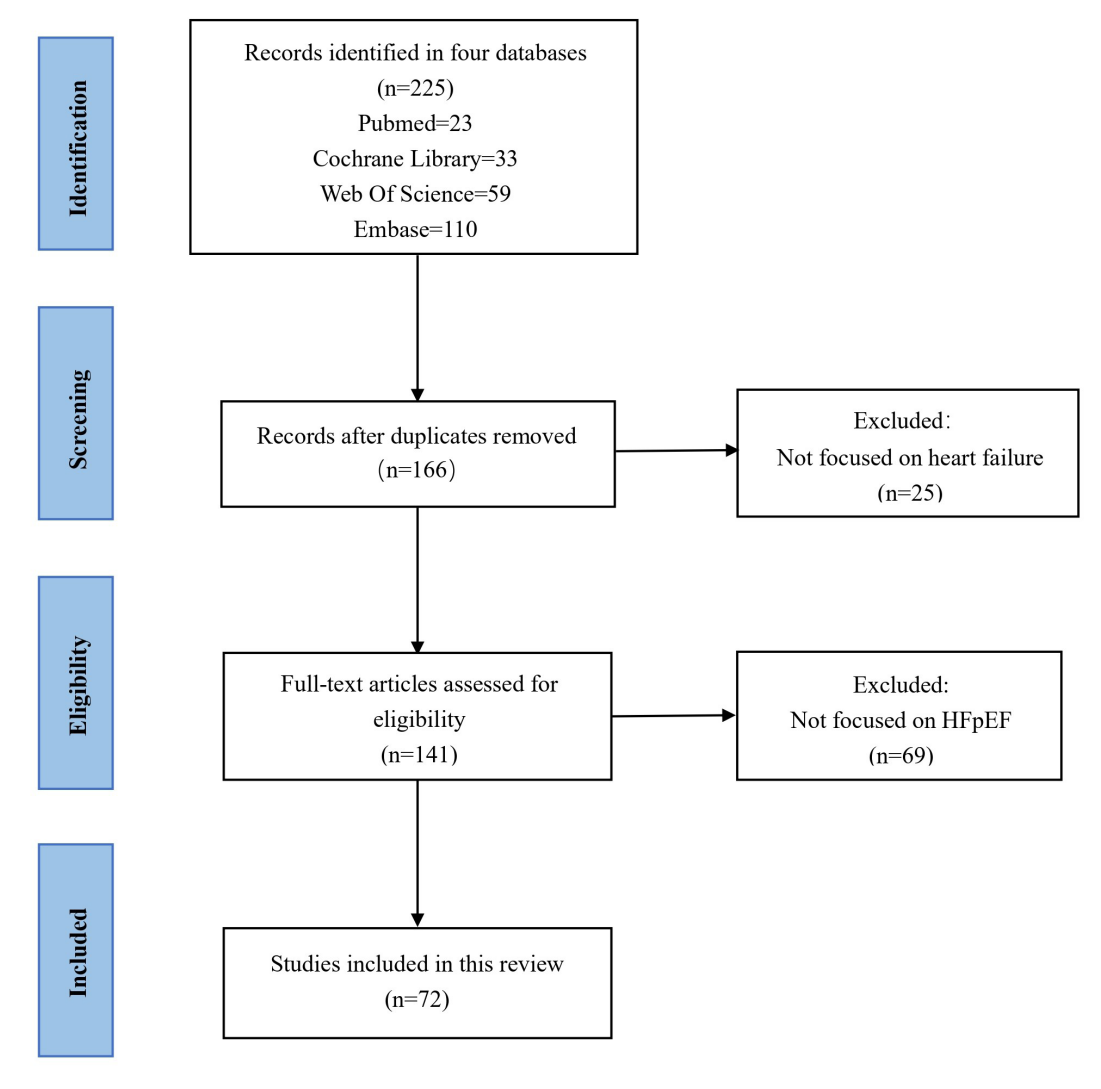
**Literature screening process**.

The initial search identified 225 articles. After the removal of duplicates, 166 records remained. Screening of titles and abstracts led to the exclusion of 25 articles that were unrelated to heart failure. Subsequently, a full-text review of the remaining articles resulted in the exclusion of an additional 69 studies, as they did not specifically address the HFpEF phenotype. Ultimately, 72 articles met the inclusion criteria and were selected for analysis. The literature screening process is summarized in the flow diagram presented in Fig. 2.

## 3. Features of Manifestations of Heart Failure With Preserved Ejection Fraction

### 3.1 Pathophysiology

**Fig. 3.**
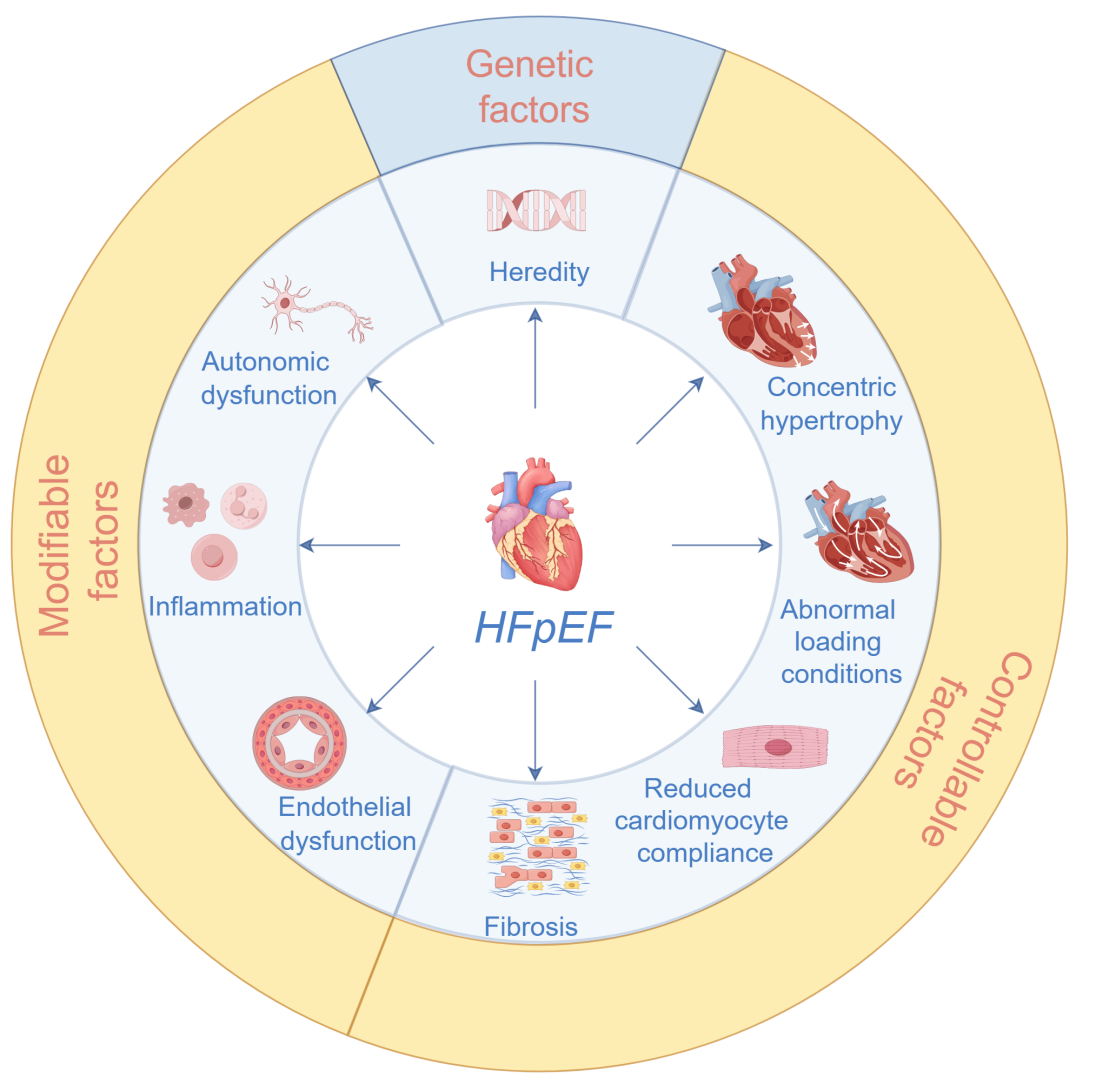
**Pathophysiological mechanisms associated with HFpEF**. The ones that fall in the yellow band (modifiable and controllable factors) form the focus in nursing practice.

HFpEF is not a precursor to HFrEF but represents a distinct phenotypic category, as clearly classified in the ESC guidelines, underpinned by different pathophysiological pathways [2]. Compared with HFrEF, HFpEF exhibits more heterogeneous mechanisms involving a complex interplay of multiple factors that collectively impair cardiac function, reflecting a multifactorial and polygenic pathology. A schematic of the key pathophysiological mechanisms associated with HFpEF is provided in Fig. 3. Notably, many of these mechanisms involve risk factors that are controllable or modifiable through nursing interventions.

In patients with HFpEF, diastolic stiffness and dysfunction of the left ventricle lead to elevated left ventricular filling pressures at rest and during exercise. This elevation increases pulmonary venous pressure, impairs alveolar gas exchange and oxygen-carrying capacity, and may ultimately result in pulmonary hypertension. Chronic elevation of left ventricular filling pressures also promotes left atrial remodeling and dysfunction [8]. Characteristic structural alterations include concentric myocardial hypertrophy and reduced ventricular cavity size, manifesting as decreased end-diastolic volume, stroke volume, and cardiac output [1, 9]. A key hallmark of HFpEF is an upward and leftward shift of the left ventricular end-diastolic pressure-volume relationship [10].

Evidence suggests that increased epicardial adipose tissue can promote a state of low-grade systemic inflammation, which enhances endothelial inflammatory activation. This process, in turn, induces alterations in myocardial elasticity, promotes collagen deposition, and drives myocardial fibrosis [8]. Myocardial fibrosis, along with these structural changes, impairs ventricular filling and reduces the efficiency of cardiac pumping, ultimately leading to low cardiac output and hypoxia at the cellular and tissue levels [9]. Furthermore, studies by Todaka *et al*. [11], using an animal model of cardiomyopathy induced by repeated coronary microembolization, identified coronary microcirculatory endothelial dysfunction as an early factor in the development of cardiac dysfunction [10]. Méndez *et al*. [1] emphasized that comorbid conditions in HFpEF contribute to a systemic pro-inflammatory state, which triggers inflammation of the coronary microvascular endothelium. They propose endothelial dysfunction as the primary pathophysiological abnormality in HFpEF [1]. Additionally, chronotropic incompetence represents another relevant mechanism, potentially related to reduced baroreflex sensitivity and increased sympathetic stimulation due to underlying autonomic dysfunction [1].

### 3.2 Epidemiological Features

Compared to patients with HFrEF, those with HFpEF are typically older, more often female [12, 13], and more likely to be obese [14]. They also exhibit a higher burden of comorbidities and frailty [3]. In a study comparing racial disparities between Black and White heart failure patients, Dickson *et al*. [15] reported that Black individuals with HFpEF were taking more medications at symptom onset and demonstrated poorer medication adherence and less adequate disease-related knowledge.

In recent years, the prevalence and hospitalization rates of HFpEF have increased significantly [16, 17], with comorbidities contributing to elevated readmission rates [12, 13]. Wang *et al*. [16] analyzed 15-year trends in the Australian Heart Failure Management Program and observed that the prevalence of HFpEF rose from 24% to 35% between 2001 and 2015. Similarly, Steinberg *et al*. [17] reported that hospitalizations for HFpEF increased from 33% to 39% from 2005 to 2010, although in-hospital mortality declined during this period.

Gotsman *et al*. [12] found that while overall hospitalization rates were similar between HFpEF and HFrEF, the causes of hospitalization differed: HFrEF patients were more frequently admitted for cardiac reasons, whereas HFpEF patients were more often hospitalized for non-cardiac conditions. This pattern is likely attributable to the older age and higher comorbidity burden in the HFpEF population. Supporting this, Li *et al*. [14] noted that non-cardiac conditions such as chronic obstructive pulmonary disease (COPD) and urinary tract infections are common reasons for hospitalization among HFpEF patients. However, a study by Daamen *et al*. [18] on heart failure phenotypes in nursing homes reported a roughly equal prevalence of HFpEF and HFrEF, with COPD being more common in HFrEF patients. Regarding outcomes, Wong *et al*. [13] indicated that patients with preserved systolic function had higher comorbidity-related readmission rates, suggesting that reduced survival in this group may be linked more to comorbidities than to inadequate cardiac management. Loop *et al*. [19] reported similar readmission rates and lengths of stay for HFpEF and HFrEF patients, but 30-day mortality was 10% lower in the HFpEF group. From a care delivery perspective, Masters *et al*. [20] highlighted that community-based services provide substantially less support for HFpEF patients compared to those with other heart failure phenotypes. Resource allocation issues may contribute to these disparities in care across different heart failure service providers [20].

These findings underscore the importance of developing personalized nursing care plans tailored to the specific needs of HFpEF patients, with particular emphasis on the formulation and implementation of comorbidity management strategies.

### 3.3 Comorbidities

Current research on comorbidities in HFpEF remains limited, with most studies focusing on the coexistence of HFpEF and COPD. Osundolire *et al*. [21] investigated the prevalence of COPD across heart failure phenotypes among nursing home residents. Their results indicated a higher prevalence of COPD in patients with unspecified types of heart failure compared to those with HFpEF or HFrEF. Additionally, individuals with multiple chronic conditions and current smokers showed a higher prevalence of COPD. Gulea *et al*. [22] analyzed clinical outcomes and healthcare resource utilization in HFpEF and HFrEF patients with comorbid COPD. Compared to HFrEF patients with COPD, those with HFpEF and COPD had a higher risk of acute COPD exacerbations, more frequent long-term hospitalizations and emergency department visits, and higher average pharmacy costs. However, they exhibited lower risks of mortality and certain cause-specific hospital admissions.

Campos *et al*. [23] examined diagnostic and treatment pathways for HFpEF patients with obesity. The findings revealed a low diagnostic rate of HFpEF by primary care providers, with nearly half of the patients being referred to specialists. The authors emphasized the need to enhance education on HFpEF diagnosis in primary care and to improve coordination between primary and specialist care to optimize patient management. Philippou *et al*. [24] evaluated treatment adherence in heart failure patients with diabetes. They reported that HFpEF patients with diabetes who received fewer heart failure medications had poorer blood pressure control compared to their HFrEF counterparts. Jia *et al*. [25] investigated the use of Sacubitril/Valsartan in patients with HFpEF and comorbid chronic kidney disease (CKD). The study results demonstrated that, compared with angiotensin-converting enzyme inhibitors (ACEI)/angiotensin receptor blockers (ARB) therapies, Sacubitril/Valsartan—even at low doses—more effectively delayed the progression of renal dysfunction and promoted reverse myocardial remodeling. However, its protective effect on urinary protein was less pronounced than that of ACEI/ARB regimens [25].

### 3.4 Risk Factors

Several risk factors contribute to the development and progression of HFpEF. Genetic predisposition is a fundamental element. Studies have shown a rising incidence of sudden cardiac death (SCD) in HFpEF patients. Genome-wide association studies (GWAS) have identified specific genetic pathways associated with HFpEF risk, onset, and progression. However, the genetic susceptibility to SCD in this population remains uncharacterized [26].

Controllable and modifiable risk factors are of particular relevance to nursing practice. Alogna *et al*. [27] reported that interleukin-6 levels are typically elevated in HFpEF and are associated with more severe symptoms, reduced exercise capacity, and increased upper body fat accumulation. Méndez *et al*. [1] highlighted that comorbidities such as obesity, coronary artery disease, hypertension, and diabetes in HFpEF promote a systemic pro-inflammatory state, leading to coronary microvascular endothelial inflammation and dysfunction. Moreover, increased body mass index is correlated with higher epicardial adipose tissue [8].

Among these factors, elevated body mass index and obesity are modifiable, while conditions such as atrial fibrillation, coronary artery disease, hypertension, and diabetes can be managed through treatment and are thus considered controllable. Clinical nursing strategies centered on HFpEF risk factor management may include health education on weight control and individualized care plans for often-overlooked comorbidities.

## 4. Clinical Practice

### 4.1 Diagnosis

Non-specific symptoms and signs, combined with frequent comorbidities, complicate the diagnosis of HFpEF, leading to underdiagnosis in up to two-thirds of cases [28]. Confidence in diagnosing HFpEF varies considerably among healthcare professionals, ranging from 58% among cardiologists to only 6% among heart failure nurses—significantly lower than for HFrEF—highlighting a widespread lack of adequate awareness among general practitioners and nurses [28].

Several novel diagnostic approaches have been proposed, though their translation into nursing practice remains limited. Bhattarai *et al*. [29] differentiated HFpEF from HFrEF by comparing symptom clusters associated with varying degrees of congestion. Méndez *et al*. [1] noted that two score-based algorithms, H_2_FPEF and HFA-PEFF, are used in HFpEF diagnosis, though both have limitations. Smeets *et al*. [30] improved diagnostic accuracy by expanding audits of electronic health record data from general practitioners, while cautioning against overdiagnosis. Tabassian *et al*. [31] applied statistical modeling and machine learning to analyze spatiotemporal changes in left ventricular strain rate at rest and during exercise, offering an objective basis for heart failure phenotyping.

The utilization of diagnostic tests also reflects varying levels of confidence across professions. Echocardiography is used by 91% of cardiologists, 67% of general practitioners, 30% of general internal medicine physicians, 21% of nurses, and 17% of general practitioner partners [28]. Cardiologists are also more likely than other providers to use electrocardiograms (ECGs) and chest X-rays for diagnosing HFpEF. In contrast, clinical assessment of symptoms and signs and natriuretic peptide testing are less commonly employed [28, 30].

Care priorities differ between undiagnosed and diagnosed HFpEF patients. Due to the non-specific clinical presentation, underdiagnosis is common [28]. Prior to diagnosis, care focuses on symptom management and supportive nursing interventions, though such support is not always accessible [32]. Therefore, a comprehensive evaluation is essential to achieve a timely and accurate diagnosis. For those diagnosed with HFpEF, a holistic, multidisciplinary approach should be adopted, including comorbidity management, congestion relief, self-management support, and referral to cardiac rehabilitation [32]. Care in this context emphasizes long-term management and complication prevention. In both scenarios, personalized treatment plans and active patient engagement in self-management are essential.

### 4.2 Treatment

Currently, there are no evidence-based treatments specifically approved for HFpEF [33]. Existing management strategies encompass both pharmacological and non-pharmacological approaches; however, most heart failure-specific treatments evaluated in randomized controlled trials have not demonstrated significant reductions in cardiovascular morbidity or mortality in the HFpEF population [34].

#### 4.2.1 Pharmacological Treatments

Many medications effective in HFrEF have not shown comparable benefits in reducing hospitalization or mortality for HFpEF patients [8]. To date, sodium-glucose cotransporter-2 (SGLT-2) inhibitors are the only class of drugs proven in randomized clinical trials to lower both hospitalization rates and cardiovascular mortality in HFpEF [8, 34]. Current guidelines recommend that nurse practitioners initiate SGLT-2 inhibitors in patients with suspected heart failure, irrespective of ejection fraction [8, 35]. Notably, glucagon-like peptide-1 receptor agonists (GLP-1 RAs), initially developed as glucose-lowering agents, have exhibited multiple pleiotropic effects beyond glycemic control. These include reducing inflammation and oxidative stress, promoting vasodilation, decreasing arterial stiffness, and attenuating myocardial fibrosis—all of which are key pathophysiological mechanisms in HFpEF. Relevant studies suggest that GLP-1 RAs may reduce the composite endpoint of cardiovascular death or worsening heart failure in HFpEF patients [36, 37]. Furthermore, semaglutide has shown efficacy in improving quality of life in obese HFpEF patients, though further clinical studies are needed to confirm its long-term efficacy and safety in the broader HFpEF population [38]. Loop diuretics can alleviate symptoms of fluid overload in HFpEF but do not significantly improve hospitalization rates or cardiovascular mortality [34]. The effects of other drug classes remain controversial, including mineralocorticoid receptor antagonists (MRAs), beta-blockers (BBs), renin-angiotensin-aldosterone system (RAAS) inhibitors, digoxin, and angiotensin receptor-neprilysin inhibitors (ARNI) [34]. Meyer *et al*. [39] compared non-dihydropyridine calcium channel blockers (non-DHP CCBs) and BBs regarding hospitalization risk in HFpEF. Results indicated a slight reduction in heart failure hospitalization among non-DHP CCBs users and lower all-cause mortality in BBs users. However, the 2022 American Heart Association heart failure guidelines, citing limited evidence and potential adverse effects (such as reduced exercise capacity), withdrew the Class IIa recommendation for BBs use in HFpEF [39]. Nanayakkara *et al*. [40] reported in a study that extended-release oral milrinone demonstrated good tolerability and was associated with improved quality of life in HFpEF patients, though longer-term studies are needed to establish the role of this therapeutic approach in HFpEF management.

The following considerations are essential in the nursing management of HFpEF pharmacotherapy (see Table 1, Ref. [34, 40, 41, 42]). SGLT-2 inhibitors lower blood glucose by promoting urinary glucose excretion and exert diuretic effects via renal sodium excretion. Nursing monitoring should include blood glucose, blood pressure, and hydration status, with particular attention to preventing urinary tract and genital infections [34, 41]. Cases of euglycemic diabetic ketoacidosis (euDKA) have been reported in HFpEF patients on SGLT-2 inhibitors, necessitating clinical vigilance [42]. Diuretics help manage fluid overload symptoms. Their use should be accompanied by close monitoring of symptoms, fluid intake and output, body weight, and electrolyte levels to guide dosage adjustments [34]. As the efficacy of other drugs in HFpEF remains uncertain, nursing staff should be attentive to potential adverse reactions. A thorough understanding of medication functions and side effects is essential for monitoring patient responses, assisting physicians in managing adverse events, and facilitating timely medication adjustments. Given that most HFpEF patients are elderly with multiple comorbidities, special attention should be paid to dosing and polypharmacy. Patients should be instructed to adhere to regular follow-up assessments of liver and kidney function, electrolyte levels, and other relevant parameters [34]. A comparison of drug treatment effects in HFpEF is provided in Table 1.

**Table 1.** **Comparison of drug treatment effects in heart failure with preserved ejection fraction**.

Category	Medication	Key efficacy outcomes	Essential nursing considerations
SGLT-2 inhibitors [34, 41, 42]	Empagliflozin, Dapagliflozin, Sotagliflozin	•Reduces hospitalizations and cardiovascular mortality •Sotagliflozin can improve emergency visit outcomes	•Monitor for signs of euDKA
			•Assess volume status and renal function routinely
			•Provide patient education on genital hygiene to prevent infections
Diuretics [34]	Loop Diuretics	•Relieves symptoms of fluid overload •Debated impact on hospitalizations and cardiovascular mortality	•Monitor daily weight, I/O balance, and symptoms of dehydration
			•Assess electrolytes (especially K^+^, Na^+^) regularly
RAAS inhibitors [34]	ACEIs, ARBs	•Debated benefit in HFpEF	•Monitor for hypotension, renal function, and hyperkalemia
ARNI [34]	Sacubitril-Valsartan	•No significant difference in hospitalization rates and cardiovascular mortality	•Ensure a 36-hour washout from ACEI/ARB before initiation
			•Monitor for angioedema, hypotension, and renal function
BBs [34]	Bisoprolol, Metoprolol	•Debated benefit in HFpEF	•Monitor heart rate, blood pressure, and signs of fatigue
			•Guide on appropriate physical activity and self-monitoring
Digitalis glycoside [34]	Digoxin	•Reduce hospitalization rates •No impact on cardiovascular mortality	•Monitor heart rate/rhythm and serum digoxin levels
			•Assess for toxicity
Phosphodiesterase inhibitor [40]	Milrinone	•Improves quality of life	•Monitor heart rate, blood pressure, body weight and volume status.
			•Assess electrolytes and renal function.

ACEIs, angiotensin-converting enzyme inhibitors; ARBs, angiotensin receptor blockers; ARNI, angiotensin receptor-neprilysin inhibitor; BBs, beta-blockers; euDKA, euglycemic diabetic ketoacidosis; RAAS, renin-angiotensin-aldosterone system; SGLT-2, sodium-glucose cotransporter 2; I/O, input/output.

#### 4.2.2 Non-Pharmacological Treatments

Non-pharmacological management of HFpEF primarily includes exercise training, device therapy, and dietary interventions. These three modalities address different aspects of the condition, each with distinct advantages and limitations. A comparison of non-pharmacological treatment options for HFpEF is provided in Table 2 (Ref. [1, 9, 34, 43, 44, 45, 46, 47, 48]).

**Table 2.** **Comparison of non-pharmacological treatment modalities in heart failure with preserved ejection fraction**.

Treatment modality	Pros	Cons	Results	Nursing considerations
Exercise training [9, 34, 43, 44]				
Combined exercise/High-intensity interval training	•Low cost	•Poor adherence	•Improves peak exercise capacity, submaximal exercise capacity, and quality of life •Increases 6-minute walk distance •Benefits atrial remodeling and left ventricular diastolic function	•Individualized patient assessment •Exercise safety education •Intensity adaptation guidance •Routine monitoring and regular reevaluation
	•Non-invasive	•Requires supervised program		
		•Limited applicability due to high intensity		
		•Risk of secondary injury		
Pedometer-based walking intervention [45]	•Low cost	•Requires long-term device wear •Device dependence		
	•Non-invasive			
	•Convenient and flexible			
	•Low risk of secondary injury			
Device therapy				
CardioMEMS [1, 34, 46, 47]	•Accurately assesses volume status	•High initial cost •Invasive procedure	•Reduces HFpEF hospitalization rates •Allows for longer effective follow-up periods	•Pre- and post-procedural patient education and psychological support •Complication prevention and monitoring •Lifestyle and home monitoring guidance •Regular follow-up scheduling
	•Reduces hospitalization costs (cost-neutral within 1 year)			
Atrial shunt device [34]	•Reduces left atrial pressure and pulmonary capillary wedge pressure	•Invasive procedure	•Not yet proven to reduce cardiovascular morbidity or mortality	
Diet therapy [48]				
Ketogenic diet therapy	•Low cost •Non-invasive	•Long-term adherence challenges	•Efficacy remains under investigation	•Individualized nutritional assessment and guidance
				•Routine monitoring and psychological support

CardioMEMS, wireless implantable pulmonary artery hemodynamic monitoring systems.

Exercise training is recognized as an important adjunct therapy in heart failure, capable of improving quality of life, enhancing peak and submaximal exercise capacity, alleviating fatigue [43], and increasing 6-minute walk distance [9, 34]. Even elderly patients with physical limitations can benefit from regular, cardiac-appropriate training [44]. Study has been conducted to compare the effects of combined exercise (aerobic plus strength training) versus high-intensity interval training on exercise capacity, diastolic function, endothelial function, and arterial stiffness in patients with HFpEF [49]. Other investigations focus on pedometer-based walking interventions and their impact on functional capacity and neurohumoral regulation [45]. Alonso *et al*. [43] have outlined plans to explore strategies for improving exercise adherence and determining optimal exercise dosing through both face-to-face and virtual coaching. While specific nursing-focused outcomes from these studies are still awaited, Sachdev *et al*. [50] have highlighted that exercise-based interventions consistently demonstrate significant and clinically meaningful improvements in symptoms, exercise capacity, and quality of life in HFpEF patients.

Device therapy for HFpEF includes wireless implantable pulmonary artery hemodynamic monitoring systems (CardioMEMS) and atrial shunt devices [51, 52]. CardioMEMS is currently the only FDA-approved wireless pulmonary artery pressure monitor and has been shown to substantially reduce HF-related hospital readmissions [46]. Elkammash *et al*. [34] reported significant reductions in hospitalization rates among HFpEF patients with CardioMEMS use. Gibson *et al*. [47] indicated that remote monitoring with CardioMEMS can improve patients’ functional class, reduce hospitalizations and emergency department visits, and lower the average economic burden on patients within one year post-implantation, despite the high initial cost. Méndez *et al*. [1] also recognized CardioMEMS as a valuable tool for assessing volume status in HFpEF. The atrial shunt device, a percutaneously implanted interatrial catheter, can reduce left atrial pressure and pulmonary capillary wedge pressure; however, no current evidence demonstrates its efficacy in reducing cardiovascular morbidity or mortality in HFpEF [34].

Evidence regarding dietary interventions in HFpEF remains limited. While some studies suggest potential benefits in preventing critical outcomes, heterogeneous methodologies preclude definitive recommendations [48]. Gonçalves *et al*. [53] conducted a study on an HFpEF rat model, indicating that providing ketone bodies through diet or supplements could serve as a highly valuable adjunctive strategy for HFpEF treatment.

Nursing considerations for these non-pharmacological approaches are as follows:

Exercise Training: Before initiation, a comprehensive patient assessment should be performed to develop an individualized exercise plan and provide safety education. Currently, no formal exercise prescription exists for unsupervised training [50]. The choice of exercise should account for the patient’s functional capacity and gait stability. Non-frail patients are advised to engage in moderate-intensity exercise 3–5 days per week, for 20–60 minutes per session. Frail patients may begin with multiple shorter sessions, gradually increasing duration and intensity [54]. Regular evaluation of cardiac function and exercise tolerance is essential for adjusting the exercise regimen.

Device Therapy: Pre-implantation, healthcare providers should assess the patient’s overall health and provide detailed explanations regarding device costs, principles, procedures, expected outcomes, and potential risks. Psychological support is also important. Post-implantation, routine monitoring of HFpEF-related symptoms and medication adherence is necessary, along with complication prevention such as wound infection and thrombosis. Patients should be advised to attend regular follow-ups for cardiac function assessment and to avoid strenuous activities or impacts to the device site. Those with CardioMEMS devices should receive education on the correct use of the external reader and be encouraged to transmit monitoring data regularly to facilitate timely treatment adjustments [55].

Dietary Intervention: As the ketogenic diet remains under investigation, dietary planning should be guided by a physician or dietitian and tailored to individual needs. Caregivers should instruct patients to maintain appropriate macronutrient ratios, supplement essential nutrients, avoid processed foods, and ensure adequate hydration. Regular monitoring of cardiac function, symptoms, and relevant laboratory parameters—such as ketone bodies, lipids, and blood glucose—is recommended, along with maintaining electrolyte balance. Psychological support should also be integrated.

### 4.3 Prognosis

Driven by population aging, the prevalence of heart failure continues to rise. Patients often experience recurrent hospital readmissions, imposing a substantial burden on both families and the healthcare system. Predicting readmissions using risk models is crucial for the efficient allocation of nursing resources. However, given the distinct pathophysiological mechanisms and treatment priorities between HFpEF and HFrEF, developing phenotype-specific readmission prediction models that better align with clinical needs remains an unmet challenge.

Sue-Ling and Jairath investigated risk factors for 31- to 60-day readmissions among elderly African American and Caucasian female heart failure patients. Their findings indicated that neither heart failure phenotype (HFrEF vs. HFpEF) nor race predicted readmission within this specific timeframe [56]. In contrast, Song *et al*. [57] identified that the ratio of mitral inflow velocity to early diastolic mitral annular velocity and the presence of depressive symptoms independently predicted readmissions in Korean patients with heart failure and preserved systolic function. Moreover, moderate to severe depressive symptoms were associated with higher readmission rates and shorter time to readmission. To prevent early readmissions, the authors emphasized the importance of implementing continuous management strategies for depressive symptoms, extending from the hospital setting to the home environment [57].

### 4.4 Management

#### 4.4.1 Controlling Etiologies and Risk Factors

The 2021 ESC heart failure guidelines recommend the control of etiologies and risk factors as a cornerstone of HFpEF management [34]. This approach is central to current clinical strategies. Key risk factors encompass hypertension, coronary artery disease, obesity, diabetes, atrial fibrillation, renal failure, and depression [1, 8, 58, 59]. Studies indicate that hypertension is among the most significant contributors to the development of HFpEF [60], and sustained management of hypertension may help prevent its onset [44, 60].

#### 4.4.2 Nursing Management Models

Nursing management models for HFpEF are diverse, and the optimal structure remains under investigation. Tran *et al*. [61] reported no significant differences in hospitalization or mortality rates between specialized HFpEF clinics and general clinics, suggesting that the structure, processes, and treatment priorities of dedicated clinics may not fully align with the actual needs of HFpEF patients. This highlights the need for quality improvement initiatives to establish more effective and comprehensive clinical pathways. Kyriakou *et al*. [62] further noted that the HFpEF population lacks evidence-based treatments and presents with multiple comorbidities, necessitating management strategies distinct from those for other heart failure phenotypes. Recent advances in HFpEF nursing models can be categorized into four areas: multidisciplinary management, cardiac rehabilitation, monitoring, and medication management. A comparison of the advantages and disadvantages of these models is provided in Table 3 (Ref. [3, 4, 43, 63, 64, 65, 66, 67]).

**Table 3.** **Comparison of heart failure with preserved ejection fraction nursing management models**.

Management model	Pros	Cons	Reported outcomes
Multidisciplinary management model [3, 66, 67]	•Comprehensive patient assessment. •Diverse expertise.	•Optimal service model not defined.	Aims for holistic care, but proven benefits are unclear.
Cardiac rehabilitation models			
Traditional center-based [4, 65]	•Concentrated medical resources. •Diverse team.	•Low participation/completion rates.	Reduces all-cause mortality and readmission.
Home-based [4]	•High participation and adherence.	•Limited medical resources.	Improves quality of life and exercise capacity.
	•Convenient; supports mental health.	•Simpler team structure.	
Monitoring models			
Self-monitoring [64]	•Increases patient engagement and knowledge.	•Relies on patient’s knowledge and self-discipline.	Inconsistent study results.
		•Optimal method not standardized.	
Remote monitoring [43]	•Efficient for large populations. •Economical and easy to use.	•Cannot replace emergency care.	Research in progress
		•Relies on technology and patient engagement.	
Medication management model			
Mnemonic aids [63]	•Aids clinical decision-making for nurses.	•Lacks specificity; must be individualized.	Supports nurse practitioners in medication management.

4.4.2.1 Medication Management Model

The management of HFpEF presents challenges for nurse practitioners due to limited pharmacological options. Research by Kato *et al*. [68] found that poor medication adherence was not an independent risk factor for adverse clinical outcomes in HFpEF patients, potentially reflecting the current scarcity of proven effective pharmacotherapies for this condition. Current pharmacological management of HFpEF focuses on controlling complications and improving quality of life. El Hussein and Blayney [63] developed a seven-letter mnemonic strategy to guide nurse practitioners in medication management for HFpEF.

4.4.2.2 Monitoring Model

Given the limited effective treatments for HFpEF, Zaharova *et al*. [64] suggested that self-management—particularly through improved symptom monitoring and treatment adherence—may be especially beneficial for these patients. Alonso *et al*. [43] are comparing face-to-face and virtual coaching to identify the most effective and cost-efficient strategies for promoting exercise adherence and determining optimal exercise dosing in HFpEF. Ansari *et al*. [69] noted that AI-guided neuromodulation could provide personalized and more effective interventions for HFpEF, though current research on AI-driven neuromodulation for HFpEF remains limited.

4.4.2.3 Cardiac Rehabilitation Model

While exercise-based cardiac rehabilitation is known to benefit HFpEF patients, participation rates remain suboptimal. Potential barriers include the absence of commercial liability coverage at rehabilitation centers, logistical challenges in attending regular hospital visits, and patient reluctance to participate in group sessions [4]. Kitagawa *et al*. [65] noted that although referral and completion rates for outpatient cardiac rehabilitation are low among HFpEF patients, those who complete the programs demonstrate favorable outcomes. Lang *et al*. [4] tested a home-based cardiac rehabilitation model delivered by healthcare professionals and focused on comprehensive self-management. Results indicated that this model is feasible and acceptable to both HFpEF patients and their caregivers, and can improve health-related quality of life and exercise capacity .

4.4.2.4 Multidisciplinary Management Model

HFpEF patients are typically older with multiple comorbidities, necessitating multidisciplinary management and interdisciplinary coordination involving comprehensive medical assessments [3, 66]. Although multidisciplinary heart failure management programs are internationally recommended, evidence of their effectiveness in HFpEF is limited, and the optimal service model remains unclear [3].

Current reports on nurse-led multidisciplinary management models remain limited. Lee’s team demonstrated that a specialist-nurse-led multidisciplinary clinic significantly reduced hospitalization rates in HFpEF patients, albeit requiring higher diuretic doses and longer clinic visits [70]. Andryukhin’s team implemented a structured nurse-led disease management program in primary care, which effectively improved emotional status, quality of life, and cardiac function while attenuating cardiac remodeling [67].

Other relevant studies on multidisciplinary management models include: Hawley *et al*. [71] developed a comprehensive heart failure service based on a multidisciplinary approach that significantly reduced 30-day mortality and HF-related readmission rates in HFpEF patients. Kearney’s team confirmed that a specialist-led model with nursing support effectively reduced hospitalization and mortality rates [72].

Nursing care is integral to all aspects of clinical management in HFpEF. Enhancing diagnostic confidence requires updated guidelines, refined clinical practices, and the development of improved diagnostic tools. Nursing staff should be proficient in the indications and side effects of HFpEF medications and monitor their use—a process that can be supported through structured medication management programs to aid clinical decision-making. As Bionat and Delaflor Santa Ana emphasize, providing targeted patient education on risk factors is essential in HFpEF care [66]. Given that the HFpEF population is predominantly older and female, with a high burden of comorbidities, such education can facilitate the implementation of healthy lifestyle interventions, improve control of underlying conditions, and enhance quality of life. Nurses should be skilled in recognizing common comorbidities and collaborate with physicians to deliver appropriate treatment. Effective comorbidity management may help reduce systemic inflammation and slow the pathophysiological progression of HFpEF. The development of risk prediction models for HFpEF readmissions enables early identification and intervention for high-risk patients, thereby reducing health risks and alleviating financial burdens. Multidisciplinary nursing management models are particularly well-suited to the complex clinical profile of HFpEF and should be implemented according to local resources and needs. Remote monitoring systems based on the Internet of Medical Things (IoMT) can track vital signs and symptom changes in HFpEF patients, allowing for the timely detection of potential issues in daily care. Patient self-management education is also crucial, empowering individuals to recognize early signs of heart failure, monitor weight fluctuations, adjust medications as instructed, and adhere to prescribed exercise regimens. Finally, significant attention should be directed toward ensuring smooth care transitions before and after hospital discharge.

## 5. Discussion

### 5.1 A Workflow Towards Comprehensive and Patient-Specific Nursing

A standardized workflow for HFpEF management can be synthesized from recent literature. For suspected cases, it is essential to utilize current diagnostic methods to confirm HFpEF and identify related comorbidities. Following diagnosis, a comprehensive and individualized management plan should be established, addressing risk factor control, comorbidity management, pharmacological and non-pharmacological treatments, application of care models, patient and caregiver education, self-management training, psychological support, and structured care transitions.

Long-term management and complication prevention should be emphasized throughout this process. Given that some patients exhibit poor medication adherence [15], nursing staff should strengthen medication management and education to ensure patients understand the purpose and importance of their prescriptions. Involving family members in daily care can enhance support systems and help patients better manage their condition.

As professional care institutions, nursing homes, and communities offer varying levels of resources [20], care delivery should be tailored to individual needs to optimize resource efficiency. Telemonitoring and home-based cardiac rehabilitation may help mitigate disparities in resource distribution, though further development of innovative care models and supportive policies is still needed. Additionally, robust risk prediction models for rehospitalization could help address current gaps in referral systems between primary and secondary care.

### 5.2 The Value of Clinical Guidelines

Only a minority of healthcare professionals across specialties consider clinical guidelines helpful for diagnosing and managing HFpEF. While some general practitioners find them useful for clinical decision-making, others—including certain GPs and heart failure nurses—feel that guidelines offer limited scope for personalizing HFpEF care. Cardiologists, in particular, often express greater confidence in their own clinical judgment than in strict adherence to guideline recommendations [28].

### 5.3 Contradictions Between Individual and Organizational Practices

Time constraints and heavy workloads pose significant barriers to effective HFpEF management. The absence of a structured referral system between primary and secondary care further exacerbates communication challenges [28]. Brooman-White *et al*. [3] examined care coordination for HFpEF patients and identified complexity in workflow, information transfer, and interdisciplinary relationships as key reasons why real-world practice often diverges from guideline recommendations. These insights can inform the design of more effective care coordination interventions. Lindberg *et al*. [73] noted that older, female, low-income, less-educated HFpEF patients with high comorbidity burdens are more likely to be managed solely in primary care and less often referred to specialists. This underscores the need for better identification of patients requiring specialist follow-up and for public health strategies to reduce referral inequities.

### 5.4 Challenges and Future Directions

Current HFpEF care faces numerous challenges. Nursing efforts should prioritize the following areas:

• Develop personalized comorbidity management strategies tailored to the HFpEF profile.

• Strengthen health education for patients and caregivers regarding comorbidities, risk factors, and self-management to reduce related readmissions.

• Enhance medication management and monitoring to improve adherence.

• Utilize predictive models for readmission risk to enable early intervention for high-risk patients.

• Develop and test nursing management models that better align with clinical and patient needs, improving both quality and efficiency of care.

• Optimize transitional care and referral processes before and after discharge.

Nursing care is vital for improving the quality of life and prognosis of HFpEF patients. Future work should focus on refining treatment plans, enhancing education, developing risk models, and exploring new care frameworks—all while ensuring the effective allocation of nursing resources to enable efficient and personalized care delivery.

### 5.5 Limitations

This review followed a systematic and transparent literature selection process in accordance with PRISMA guidelines. A critical narrative synthesis was performed, with careful discussion of the strengths and limitations of key evidence. However, the inability to conduct a formal quality assessment of included studies remains a limitation of this review.

## 6. Conclusion

HFpEF presents a complex clinical syndrome characterized by multifactorial pathophysiology, a high burden of comorbidities, and heterogeneous patient phenotypes. Current evidence underscores the absence of standardized nursing protocols for its diagnosis, treatment, and long-term management. To address these challenges, several key priorities in clinical nursing practice have emerged:

First, the development of individualized care plans grounded in comprehensive patient assessment is essential. Such plans should integrate control of modifiable risk factors—such as hypertension, obesity, and diabetes—along with tailored management of non-cardiac comorbidities.

Second, optimizing treatment adherence and patient self-management capabilities through structured education and continuous monitoring represents a critical nursing role. This includes medication guidance, symptom recognition, and the promotion of physical activity within safe limits.

Third, the adoption of innovative nursing models—such as multidisciplinary team care, telemonitoring systems, and home-based rehabilitation—can significantly enhance care continuity and patient outcomes, especially in resource-limited settings.

Finally, future efforts should focus on refining risk prediction tools and strengthening transitional care processes. Through the implementation of personalized, integrated, and model-supported nursing strategies, it is possible to meaningfully improve quality of life and clinical prognosis for patients living with HFpEF.
